# Hypoxia reduces CD138 expression and induces an immature and stem cell-like transcriptional program in myeloma cells

**DOI:** 10.3892/ijo.2013.2134

**Published:** 2013-10-10

**Authors:** YAWARA KAWANO, YOSHITAKA KIKUKAWA, SHIHO FUJIWARA, NAOKO WADA, YUTAKA OKUNO, HIROAKI MITSUYA, HIROYUKI HATA

**Affiliations:** 1 Department of Hematology, Kumamoto University School of Medicine, Kumamoto 860-8556, Japan; 2 Division of Informative Clinical Science, Kumamoto University School of Medicine, Kumamoto 860-8556, Japan

**Keywords:** myeloma, hypoxia, CD138, ATRA

## Abstract

Although CD138 expression is a hallmark of plasma cells and myeloma cells, reduced CD138 expression is occasionally found. However, the mechanisms underlying CD138 downregulation in myeloma cells remain unclear. Previous reports suggest that the bone marrow microenvironment may contribute to CD138 downregulation. Among various factors in the tumor microenvironment, hypoxia is associated with tumor progression, poor clinical outcomes, dedifferentiation and the formation of cancer stem cell niches in solid tumors. Since recent findings showed that progression of multiple myeloma (MM) delivers hypoxia within the bone marrow, we hypothesized that CD138 expression may be regulated by hypoxia. In the present study, we examined whether the expression of CD138 and transcription factors occurred in myeloma cells under hypoxic conditions. MM cell lines (KMS-12BM and RPMI 8226) were cultured under normoxic or hypoxic conditions for up to 30 days. Changes in the phenotype and the expression of surface antigens and transcription factors were analyzed using flow cytometry, RT-PCR and western blotting. All-trans retinoic acid (ATRA) was used to examine the phenotypic changes under hypoxic conditions. The expression levels of CD138, CS1 and plasma cell-specific transcription factors decreased under hypoxic conditions, while those of CD20, CXCR4 and B cell-specific transcription factors increased compared with those under normoxic conditions. Stem cell-specific transcription factors were upregulated under hypoxic conditions, while no difference was observed in ALDH activity. The reduced CD138 expression under hypoxic conditions recovered when cells were treated with ATRA, even under hypoxic conditions, along with decreases in the expression of stem cell-specific transcription factor. Interestingly, ATRA treatment sensitized MM cells to bortezomib under hypoxia. We propose that hypoxia induces immature and stem cell-like transcription phenotypes in myeloma cells. Taken together with our previous observation that decreased CD138 expression is correlated with disease progression, the present data suggest that a hypoxic microenvironment affects the phenotype of MM cells, which may correlate with disease progression.

## Introduction

Multiple myeloma (MM) is characterized by clonal expansion of malignant plasma cells in the bone marrow. Although novel therapeutic agents and stem cell transplantation have improved the survival of MM patients (1), MM remains an incurable disease.

Cancer stem cells are often considered to contribute to relapse and drug resistance in various cancers (2). Matsui *et al* (3) reported that myeloma stem cells are enriched in the CD138-negative population. During normal B-cell development, abundant CD138 (also known as syndecan-1: SDC1) expression is highly specific for terminally differentiated plasma cells in the bone marrow (4). Since CD138 expression is also a hallmark of malignant plasma cells (myeloma cells), it has been used for myeloma cell purification (5) and is considered to be a target for treatment (6). While the majority of myeloma cells express CD138, decreased expression of CD138 is occasionally found in clinical practice (7–9). Although the association between CD138 expression and myeloma stem cells remains a matter of debate (10), several reports have shown that CD138-low or -negative myeloma cells may contribute to drug resistance or relapse of the disease (9,11,12). Therefore, analysis of CD138 downregulation in myeloma cells is required for a better understanding of myeloma biology.

Previous reports have indicated that the bone marrow microenvironment may contribute to CD138 downregulation (13–16). Among various factors in the tumor microenvironment, hypoxia is one of the important factors associated with tumor progression, poor clinical outcomes, dedifferentiation, and formation of cancer stem cell niches in solid tumors (17). Based on recent findings showing a correlation of MM at the advanced stage with hypoxic conditions in the microenvironment within the bone marrow (18), we hypothesized that CD138 expression may be influenced by hypoxia.

In the present study, we compared the changes in CD138 and various transcription factor expressions in myeloma cells under hypoxic or normoxic conditions. We also attempted to revert CD138 expression in cells under hypoxia by treatment with all-trans retinoic acid (ATRA). The influence of ATRA on the sensitivity to bortezomib under hypoxic conditions was also examined.

## Materials and methods

### Cell culture

Human myeloma cell lines, KMS-12BM (19) and RPMI 8226 (20), were obtained from the Health Science Research Resources Bank (Osaka, Japan) and maintained in RPMI-1640 medium supplemented with 10% heat-inactivated fetal bovine serum at 37°C under 5% CO_2_. The two myeloma cell lines were cultured under normoxic (21% O_2_) and hypoxic (1% O_2_) conditions for up to 30 days, with fresh medium provided every 3 days. Experiments under hypoxic conditions were performed in a Personal CO_2_ Multigas Incubator (ASTEC, Fukuoka, Japan).

### Flow cytometric analysis of surface antigens

MM cell lines cultured under normoxic and hypoxic conditions were stained with the following fluorescently-labeled antibodies: FITCCD138 (clone MI15), FITC-CD38 (clone HIT2), PE-CD44 (clone 515), PE-CD45 (clone HI30), FITC-CD49d (clone gf10) (BD Biosciences, Franklin Lakes, NJ, USA); PE-CD54 (clone HCD54), PE-CXCR4 (clone 12G5), PE-MDR-1 (clone UIC2), APC-ABCG2 (clone 5D3) (Biolegend, San Diego, CA, USA); FITC-CD19 (clone HD37), FITC-CD20 (clone B-Ly1) (Dako, Glostrup, Denmark); and Alexa 647-CS1 (clone 162) (AbD Serotec, Oxford, UK). Density gradient centrifugation using Ficoll-Paque Plus (GE Healthcare, Uppsala, Sweden), the forward/side scatter profile and 7-amino-actinomycin D (7-AAD) (BD Biosciences) labeling were used for exclusion of non-viable cells. Flow cytometric anal ysis was performed using a FACSCalibur or FACSVerse flow cytometer (Becton-Dickinson, San Jose, CA, USA).

### Adhesion to type-1 collagen

MM cells were plated in quadruplicate at a concentration of 5×10^5^ cells/ml on type-1 collagen-coated 96-well plates (Becton-Dickinson) and incubated for 1 h at 37°C. After the incubation, the cells were washed twice with PBS and incubated with the WST-8 reagent (Dojindo, Kumamoto, Japan). The ratios of adherent cells to total applied cells were quantified by the light absorbance of each well at 450 nm using a VMax absorbance microplate reader (Molecular Devices, Sunnyvale, CA, USA).

### cDNA synthesis and reverse transcription-polymerase chain reaction (RT-PCR)

RNA was extracted from the MM cell lines using TRIzol reagent (Invitrogen, Carlsbad, CA, USA). cDNA synthesis was performed using a SuperScript III First-Strand Synthesis System for RT-PCR (Invitrogen) according to the manufacturer’s protocol.

The expression levels of *BCL6, PAX5, Oct-4, NANOG* and *SOX2* were determined by RT-PCR. β*-actin (ACTB)* was used as a normalization control. The primers for *BCL6* and *PAX5* were described previously (9). The primers for *Oct-4, NANOG, SOX2* and *ACTB* were as follows: *Oct-4* (forward, 5′-AGCC CTCATTTCACCAGGCC-3′; reverse, 5′-TGGGACTCCTCCG GGTTTTG-3′); *NANOG* (forward, 5′-ACTGTCTCTCCTCT TCCTTC-3′; reverse, 5′-CCTGTTTGTAGCTGAGGTTC-3′); *SOX2* (forward, 5′-ACAACTCGGAGATCAGCA-3′; reverse, 5′-GCAGCGTGTACTTATCCTTC-3′); *ACTB* (forward, 5′-GGACTTCGAGCAAGAGATGG-3′; reverse, 5′-AGCAC TGTGTTGGCGTACAG-3′).

Quantitative real-time RT-PCR was performed using Assay-on-Demand primers and TaqMan Universal PCR Master Mix Reagent (Applied Biosystems, Foster City, NJ, USA). Samples were analyzed using an ECO™ Real-Time PCR System (Illumina, San Diego, CA, USA). The ΔΔCt method was used to analyze the relative changes in gene expression as previously described (21), using *ACTB* as a normalization control. The following primers and probes were used: *SDC1* (Hs00896423_m1); *IRF4* (Hs01056534_m1); *PRDM1* (Hs00153357_m1); *XBP1* (Hs00964360_m1); and *ACTB* (Hs99999903_m1).

### Intracellular staining of IRF4 followed by flow cytometric analysis

The MM cell lines cultured under normoxic or hypoxic conditions were stained with an FITC-CD138 antibody (clone MI15; BD Biosciences), fixed and permeabilized using a FOXP3 Staining Buffer Set (eBioscience, San Diego, CA, USA), and then stained intracellularly with an Alexa 647-IRF4 antibody (clone 3E4; eBioscience) according to the manufacturer’s protocol. Flow cytometric analysis was performed using the FACSCalibur (Becton-Dickinson).

### Western blot analysis

Cell lysates were prepared as reported previously (22). Quantification of total protein was performed using a Pierce BCA Protein Assay Kit (Thermo Scientific, Waltham, MA, USA), and equal amounts of protein were used for analysis. The cell lysates were separated in NuPAGE Bis-Tris precast gels (Invitrogen) and transferred to PVDF membranes using an iBlot Dry Blotting system (Invitrogen). The membranes were blocked with 5% non-fat dry milk for 1 h at room temperature, followed by incubation with primary antibodies at 4°C for 18 h. The primary antibodies against HIF-1α, HIF-2α, NANOG, and SOX2 were purchased from Cell Signaling Technology (Beverly, MA, USA), while those against Oct-4, RARα, and actin were obtained from Santa Cruz Biotechnology (Santa Cruz, CA, USA). The membranes were then incubated with horseradish peroxidase-conjugated secondary antibodies (GE Healthcare) for 1 h at room temperature. Antibody-bound proteins were visualized using the ECL prime western blotting detection reagent (GE Healthcare) and an LAS-1000 bio-image analyzer (GE Healthcare).

### Aldehyde dehydrogenase (ALDH) activity

The ALDH activities of the MM cell lines cultured under normoxic and hypoxic conditions were analyzed using Aldefluor (Stem Cell Technologies, Vancouver, Canada). After adding activated Aldefluor reagent to the cell cultures, half of the cells were transferred to tubes containing an ALDH inhibitor, diethylaminobenzaldehyde (DEAB), to confirm specificity of the reagent. Samples were incubated at 37°C for 30 min and analyzed using the FACSCalibur (Becton-Dickinson).

### Analysis of apoptosis

The MM cell lines were incubated in the presence of 1 *μ*M ATRA (Sigma, St. Louis, MO, USA) or 5 nM bortezomib (Sigma) for 24 h. Apoptosis in the MM cell lines was quantified by staining with Annexin V (MBL, Nagoya, Japan) and 7-AAD (BD Biosciences). The samples were analyzed by flow cytometry (FACSVerse; Becton-Dickinson).

### Statistical analysis

The data were analyzed statistically by Student’s t-test using GraphPad Prism version 5.0 (GraphPad Software, La Jolla, CA, USA). P-values of <0.05 were considered statistically significant.

## Results

### Hypoxia reduces CD138 expression in MM cell lines

**Figure 1.**
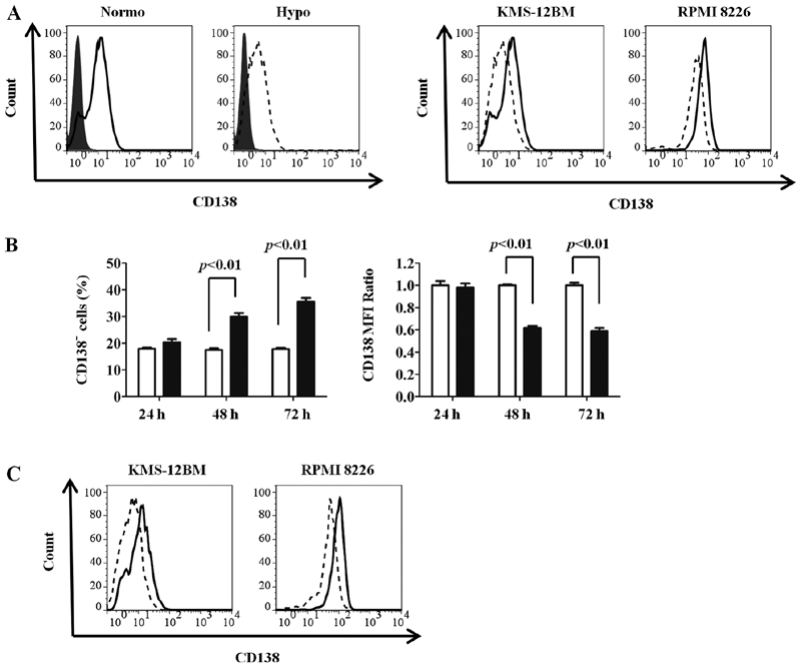
Decrease in CD138 expression under hypoxia. (A) CD138 expression in the MM cell lines is decreased after 72 h of culture under hypoxic conditions. The left panel shows CD138 expression in KMS-12BM cells under normoxic and hypoxic conditions analyzed by flow cytometry (solid or dashed line, CD138; shadowed area, isotype control). Overlay plots of the CD138 expressions in KMS-12BM and RPMI 8226 cells under normoxic (solid line) and hypoxic (dashed line) conditions are shown in the right panel. (B) CD138 expression in KMS-12BM cells is decreased in a time-dependent manner. The increasing proportion of CD138-negative cells and decrease in CD138 fluorescence intensity as judged by the mean fluorescence intensity (MFI) ratio to the isotype control are shown in the left and right panels, respectively. White bars, normoxic conditions; black bars, hypoxic conditions. (C) Re-oxygenation recovers CD138 expression. KMS-12BM cells were cultured under hypoxic conditions for 72 h. The cells were then further cultured under normoxic (solid line) or hypoxic (dashed line) conditions for an additional 72 h.

To investigate whether CD138 expression in MM cells was influenced by oxygen levels, we cultured two MM cell lines (KMS-12BM and RPMI 8226) under normoxic and hypoxic conditions for up to 72 h and compared the surface CD138 expressions by flow cytometry. Since CD138 was reported to be downregulated in non-viable cells (23), only viable cells were obtained by Ficoll density gradient centrifugation followed by gating of live cells in combination with the forward/side scatter profile and exclusion of 7-AAD-positive cells. CD138 expression was reduced by 72 h of culture under hypoxic conditions compared with normoxic conditions in both cell lines (Fig. 1A). As shown in Fig. 1B, CD138 expression was reduced from 48 h, and subsequently proceeded in a time-dependent manner. We then re-oxygenized the cells at 72 h after starting the hypoxic conditions and maintained the cells under normoxic conditions for an additional 72 h. Interestingly, we observed recovery of the CD138 expression, indicating that the reduction in CD138 expression under hypoxia was a reversible phenomenon (Fig. 1C).

### Changes in CD138 and other surface antigens under long-term hypoxia

**Figure 2.**
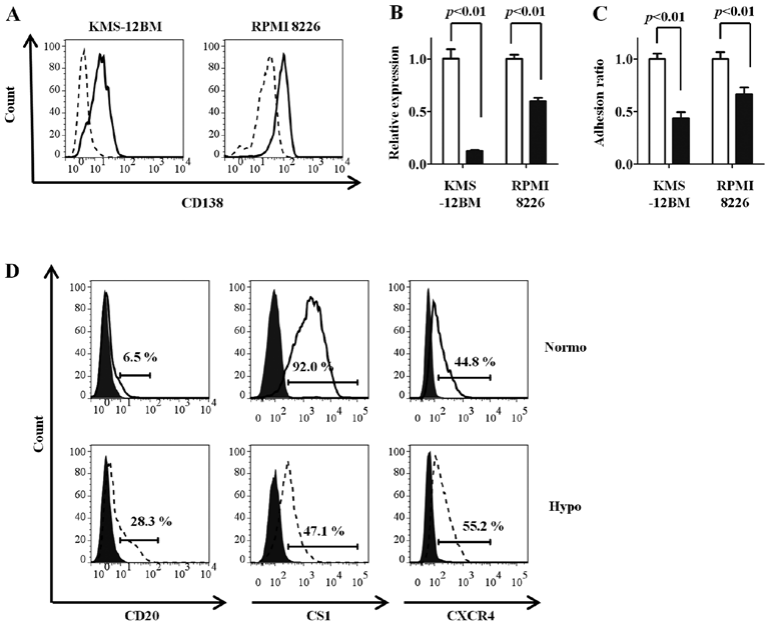
Decrease in CD138 expression and changes in other surface antigens after long-term exposure to hypoxia. (A) KMS-12BM and RPMI 8226 cells were cultured under hypoxic or normoxic conditions for 30 days and the expression of CD138 was analyzed by flow cytometry. Overlay plots of CD138 expression are shown. Normoxic conditions, solid line; hypoxic conditions, dashed line. (B) A decrease in CD138 expression is found at the mRNA level. The expression of CD138 mRNA was quantified by real-time RT-PCR. (C) Decrease in adhesion to collagen type-I under hypoxic conditions. Cells were treated as described in (A) and their adhesion capabilities to collagen type-I were analyzed. White bars, normoxic conditions; black bars, hypoxic conditions. (D) Increases in CD20 and CXCR4 expressions and decrease in CS1 expression under hypoxic conditions. KMS-12BM cells were treated as described in (A) and other surface antigens were analyzed by flow cytometry (solid or dashed line, CD138; shadowed area, isotype control).

We further analyzed CD138 expression under long-term exposure (30 days) to hypoxic or normoxic conditions. After 30 days of incubation, CD138 expression was markedly reduced in KMS-12BM and RPMI 8226 cells cultured under hypoxia compared with those cultured under normoxia (Fig. 2A). In KMS-12BM cells in particular, the positivity for CD138 expression under hypoxic conditions was reduced to only 10%. Real-time RT-PCR showed that CD138 (SDC1) mRNA was downregulated after long-term hypoxia, indicating that CD138 expression was reduced at the gene transcription level (Fig. 2B).

Since CD138 mediates the adhesion of MM cells to type-1 collagen (24), which is an important component of the bone marrow microenvironment, the influence of oxygen concentrations on the adhesion of MM cells was evaluated. The hypoxic MM cell lines adhered poorly to type-1 collagen, reflecting the low expression of CD138 (Fig. 2C).

The expression changes in other surface antigens were compared between the hypoxic and normoxic MM cell lines. The CD20 and CXCR4 expressions were increased, while the CS1 expression was decreased, under hypoxia compared with normoxia in KMS-12BM cells (Fig. 2D). Similar results were observed in RPMI 8226 cells (normoxia vs. hypoxia: CD20, 1.8 vs. 14.1%; CS1, 81.3 vs. 34.6%; CXCR4, 40.2 vs. 82.1%). No changes in expression were observed for CD19, adhesion molecules other than CD138 (CD44, CD49d and CD54), and ATP-binding cassette transporters (ABCG2 and MDR1) (data not shown).

### Hypoxic MM cell lines have an immature phenotype

**Figure 3.**
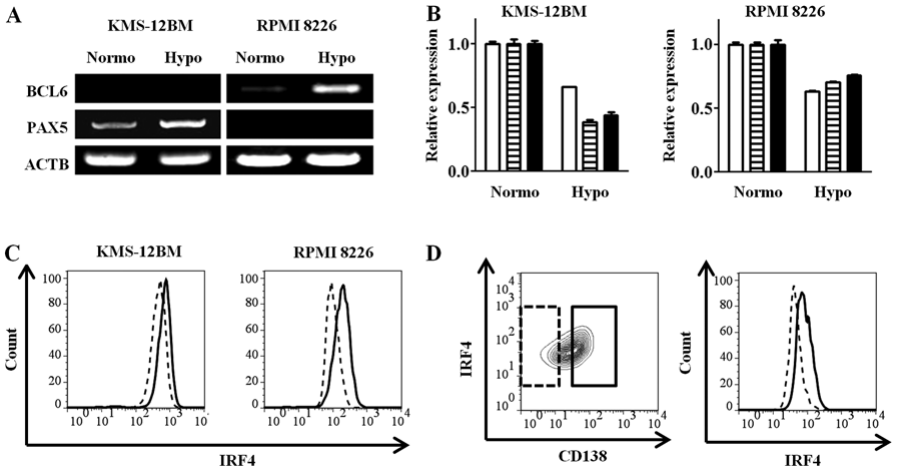
Exposure to hypoxia induces transcription factors specific for the B-cell phenotype. (A) Induction of BCL6 and PAX5 by exposure to hypoxia. KMS-12BM and RPMI 8226 cells were cultured under hypoxic conditions for 30 days. *PAX5* expression is increased in hypoxic KMS-12BM cells, while BCL6 is increased in hypoxic RPMI 8226 cells. (B) Hypoxia reduces plasma cell-specific transcription factor expression. The gene expression levels of *IRF4* (white bars), *PRDM1* (striped bars), and *XBP1* (black bars) were analyzed by real-time RT-PCR in KMS-12BM and RPMI 8226 cells. The expression of the three genes are decreased. (C) Reduction in intracellular IRF4 expression under hypoxic conditions detected by flow cytometry. The expression is decreased in hypoxic cells (dashed line) compared with normoxic cells (solid line). (D) The CD138-low subpopulation has lower IRF4 expression. RPMI 8226 cells were cultured under hypoxic conditions for 30 days. The expression of IRF4 was evaluated by gating the CD138-high (25% from the highest intensity; solid square in the left panel) and CD138-low (25% from the lowest intensity; dashed square in the left panel) subpopulations. The intracellular IRF4 expression in each compartment is shown in the right panel. The CD138-low population has lower intracellular IRF4 expression.

Since CD138 (4) and CS1 (25) expressions are highly specific for terminally differentiated plasma cells, we hypothesized that the hypoxic MM cell lines have a less mature phenotype than the normoxic cell lines. To prove this hypothesis, we assessed the expressions of the BCL6 and PAX5 transcription factors, which exist in the mature B-cell state and are decreased in mature plasma cells (26), by RT-PCR in the MM cell lines after 30 days of culture under hypoxic or normoxic conditions. Each of the mature B-cell transcription factors, *PAX5* and *BCL6*, was increased in the hypoxic state in KMS-12BM and RPMI 8226 cells (Fig. 3A). The gene expressions of *IRF4, PRDM1* and *XBP1*, which are plasma cell-specific transcription factors, were decreased in the hypoxic cells compared with the normoxic cells (Fig. 3B). The intracellular IRF4 protein expression, which was analyzed by flow cytometry, was decreased in the hypoxic cells (Fig. 3C), consistent with the results for the gene expressions. Analysis of subpopulations obtained as CD138-high and -low cells showed high and low IRF4 expressions, respectively (Fig. 3D). These findings show that the hypoxic MM cell lines with low CD138 expression have an immature, so-called mature B cell-like rather than plasma cell, transcriptional status.

### Hypoxia induces a stem cell-like transcriptional program in MM cells

**Figure 4.**
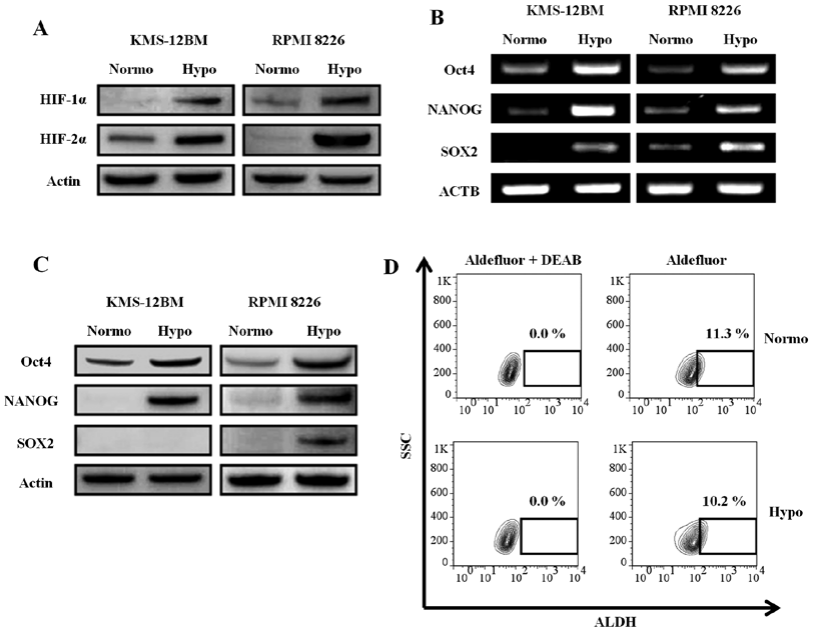
Hypoxia induces a stem cell-like transcriptional program in MM cell lines. (A) KMS-12BM and RPMI 8226 cells were cultured under hypoxic or normoxic conditions for 30 days. Cell lysates were extracted and subjected to western blot analysis. HIF expression is increased in the hypoxic MM cell lines. (B and C) Expression of transcription factors related to the stem cell phenotype was analyzed by RT-PCR (B) or western blotting (C). Upregulation of transcription factors related to the stem cell phenotype is observed under hypoxia. (D) Analyses of ALDH activity in KMS-12BM cells. Cells were cultured under hypoxic or normoxic conditions and subjected to ALDH assays. DEAB was used as an ALDH inhibitor. No increase in ALDH activity is observed in hypoxic KMS-12BM cells.

Hypoxia-inducible factors (HIFs) are key molecules for the cellular response to hypoxia. It was reported that a hypoxic environment and HIF activity are required not only for stem cells, but also for cancer stem cells (27). Since HIFs induce stem cell transcription factors in solid tumors (28), we analyzed the expression of HIFs and stem cell transcription factors in the hypoxic MM cell lines. HIFs, especially HIF-2α, were increased in the hypoxic MM cell lines, as evaluated by western blotting, indicating that the cells were actually responding to the hypoxic environment (Fig. 4A). The expression of stem cell transcription factors (Oct-4, SOX2, and NANOG) was increased in the hypoxic MM cell lines at both the mRNA (Fig. 4B) and protein (Fig. 4C) levels. The only exception was the expression of SOX2 protein, which was not detected in hypoxic KMS-12BM cells, although its mRNA expression was upregulated. However, the ALDH activity, which is characteristic of cancer stem cells including MM stem cells (29), was not increased in hypoxic KMS-12BM cells (Fig. 4D) and RPMI 8226 cells (normoxia vs. hypoxia: 1.06 vs. 0.10%). The expression of ABCG2, which is highly expressed in stem cells and associated with side-population cells (30), was not increased in hypoxic cells, as described earlier in this report.

### ATRA induces differentiation of hypoxic MM cells and increase their sensitivity to bortezomib

**Figure 5.**
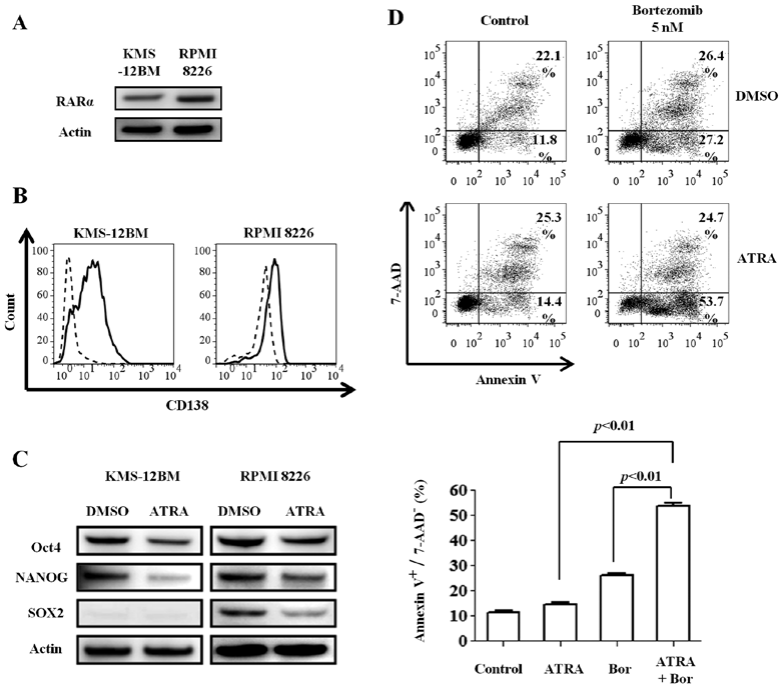
ATRA restores the phenotypic changes and sensitivity to bortezomib induced by hypoxic conditions. (A) Western blot analysis showing the expression of RARα in both KMS-12BM and RPMI 8226 cells cultured under hypoxic conditions. (B) Recovery of CD138 expression after ATRA treatment in the MM cell lines under hypoxic conditions. Solid line, ATRA treatment for 48 h; dashed line, DMSO treatment. (C) Reductions in stem cell-related transcription factors after ATRA treatment in hypoxic MM cell lines evaluated by western blot analysis. (D) Analysis of cytotoxicity toward KMS-12BM cells cultured under hypoxic conditions with ATRA and bortezomib. Cells were treated with ATRA or bortezomib alone or in combination for 24 h. The cells were then subjected to Annexin V/7-AAD analysis. The upper panel shows the raw data of the scatter graph. The lower panel shows the proportion of early apoptotic cells (Annexin V^+^/7-AAD^−^). ATRA augments early apoptosis in combination with bortezomib.

ATRA induces cell differentiation not only of stem cells (31), but also of MM cells (32,33). ATRA is also known to repress the expression of stem cell transcription factors, such as Oct-4, NANOG and SOX2 (34,35). Based on these previous reports, we investigated whether ATRA could induce redifferentiation of the hypoxic MM cell lines. We first confirmed the expression of RARα, a specific receptor for retinoic acid, in the MM cell lines cultured for 30 days under hypoxic conditions (Fig. 5A). The cell lines were then incubated with 1 *μ*M ATRA or dimethyl sulfoxide (DMSO) for 48 h under hypoxic conditions. Surprisingly, ATRA increased the CD138 expression compared with DMSO (Fig. 5B), especially in KMS-12BM cells, while the expression of all stem cell transcription factors was decreased (Fig. 5C). These findings indicate that ATRA can induce the redifferentiation of immature MM cells, even under hypoxic conditions.

Next, we investigated whether the differentiation induced by ATRA can sensitize the immature and hypoxic cells to bortezomib. Hypoxic KMS-12BM cells were treated with 1 *μ*M ATRA and 5 nM bortezomib alone or in combination for 24 h. While ATRA alone slightly affected the viability of hypoxic KMS-12BM cells, the combination of the two reagents increased the cytotoxicity, especially the early apoptosis (Annexin V^+^/7-AAD^−^ cells) (Fig. 5D).

## Discussion

Accumulating evidence indicates that CD138 is an important molecule not only for the identification of plasma cells, but also for distinguishing myeloma stem cells. CD138-negative cells have recently been proposed as myeloma stem cells (3). On the other hand, Jakubikova *et al* (10) showed that the cells in the side population analyzed by flow cytometry, which are considered to be cancer stem cells, are CD138-positive cells with clonogenic potential, while Hosen *et al* (36) reported that both CD138-positive and -negative lesions have the capacity to propagate MM *in vivo*. Although it remains unclear whether CD138-negative myeloma cells are ‘myeloma stem cells’, several reports have shown that CD138-low or -negative cells may contribute to drug resistance or relapse of the disease (9,11,12). Therefore, these previous findings allow us to consider that the expression of CD138 in myeloma cells is not uniformly high. Analyses of the mechanisms underlying the regulation of CD138 expression in myeloma cells may lead to a better understanding of myeloma biology.

In the present study, we found that a reduced oxygen level contributes to CD138 downregulation in MM cells. We observed a reduction in the CD138 level under hypoxia in a time-dependent manner that lasted for at least 30 days. The recovery of CD138 expression by re-oxygenation indicates that the oxygen level plays a major role in MM cell regulation of CD138 expression.

When the expression of CD138 was decreased, the adhesion of the MM cell lines to type-1 collagen was also decreased, indicating a correlation between CD138 expression and adhesion of MM cells to the extracellular matrix. Indeed, several reports have shown that CD138 downregulation may contribute to a more metastatic potential not only in solid tumors (37,38), but also in MM (12,39). It can be hypothesized that hypoxia may promote the metastasis of myeloma cells partly through CD138 downregulation. CXCR4, which is induced under hypoxia and contributes to migration and homing of MM cells (18), was upregulated in hypoxic MM cells. This finding may also be associated with tumor progression under hypoxia.

Hypoxic MM cells had lower CD138 and CS1 expressions and higher CD20 expression than normoxic cells. Since the expressions of CD138 (4) and CS1 (25) are highly specific for terminally differentiated plasma cells and previous reports showed an immature transcriptional profile in MM cells with low expression of CD138 (9,11,16), we hypothesized that the hypoxic MM cell lines may have a less mature phenotype than the normoxic cell lines. Expression analyses of transcription factors associated with plasma cells (IRF4, PRDM1 and XBP1) and B cells (BCL6 and PAX5) revealed that the hypoxic MM cells had a relatively immature (B cell-like) transcriptional profile compared with normoxic cells. This indicates that the oxygen levels influence not only the surface antigens, but also the transcription factor expressions in MM cells. Although hypoxic cells acquired a B cell-like phenotype, CD19 expression was negative. Since CD19 expression can distinguish MM cells from B cells and normal plasma cells (40), this indicates that hypoxia delivers an immature phenotype to MM cells, but they are not identical to normal mature B cells and can be distinguished as malignant plasma cells in terms of CD19 negativity.

HIFs (HIF-1α and HIF-2α) mediate the cellular response to hypoxia and activate transcription factors that control stem cell self-renewal (27). It was reported that HIFs are positive in myeloma cells in the bone marrow (41,42), suggesting that myeloma cells are also hypoxic *in vivo*. Our analyses of stem cell transcription factors revealed that hypoxia induces the expressions of stem cell transcription factors at both the mRNA and protein levels. Previous reports showing stem cell marker expression in CD138-negative myeloma cells (29,43) support our findings. It was also reported that Oct4 and SOX2 expression is essential for maintenance of the side-population fraction in MM (44). These findings indicate that hypoxia induces a stem cell-like phenotype in MM cells. However, we did not observe any increases in ALDH activity or ABCG2 expression, which are associated with cancer stem cell function. Taken together, hypoxia induces stem cell-like features in MM cells, although further analyses are needed to conclude that MM cells under hypoxic conditions are cancer stem cells.

It is known that MM patients with SOX2 expression have a worse overall survival than patients without SOX2 expression (45), and this report is compatible with our previous finding that MM patients with low CD138 expression showed a poor prognosis (9). MM cells under hypoxic conditions may become less sensitive to anticancer agents, thereby contributing to a poor prognosis through the acquisition of drug resistance. Indeed, ATRA was able to sensitize MM cells to bortezomib under hypoxic conditions by inducing a more differentiated phenotype. These findings allow us to propose a new therapeutic approach against CD138-low myeloma cells with drug resistance by combining ATRA with other anti-myeloma reagents such as bortezomib.

Taken together, the present data suggest that hypoxia reduces CD138 expression and provides stem cell-like features to myeloma cells. Further analyses of the mechanisms that regulate CD138 expression and related biological processes including cell adhesion or drug sensitivity should contribute not only to a better understanding of the disease, but also to an improvement of the prognosis of myeloma.
